# Effects of Added Organic Matter and Water on Soil Carbon Sequestration in an Arid Region

**DOI:** 10.1371/journal.pone.0070224

**Published:** 2013-07-16

**Authors:** Liming Lai, Yufei Li, Yuan Tian, Lianhe Jiang, Xuechun Zhao, Linhai Zhu, Xi Chen, Yong Gao, Shaoming Wang, Yuanrun Zheng, Glyn M. Rimmington

**Affiliations:** 1 Key Laboratory of Resource Plants, Beijing Botanical Garden, West China Subalpine Botanical Garden, Institute of Botany, Chinese Academy of Sciences, Xiangshan, Beijing, China; 2 Institute of RS and GIS, Peking University, Beijing, China; 3 Xinjiang Institute of Ecology and Geography, Chinese Academy of Sciences, Urumqi, Xinjiang, China; 4 State Key Laboratory of Vegetation and Environmental Change, Institute of Botany, Chinese Academy of Sciences, Beijing, China; 5 Inner Mongolia Agricultural University, Hohhot, China; 6 Key Laboratory of Oasis Ecological Agriculture of Xinjiang Bingtuan, Shihezi, Xinjiang, China; 7 Global Learning Office, College of Liberal Arts and Sciences, Wichita State University, Wichita, Kansas, United States of America; Wageningen University, The Netherlands

## Abstract

It is generally predicted that global warming will stimulate primary production and lead to more carbon (C) inputs to soil. However, many studies have found that soil C does not necessarily increase with increased plant litter input. Precipitation has increased in arid central Asia, and is predicted to increase more, so we tested the effects of adding fresh organic matter (FOM) and water on soil C sequestration in an arid region in northwest China. The results suggested that added FOM quickly decomposed and had minor effects on the soil organic carbon (SOC) pool to a depth of 30 cm. Both FOM and water addition had significant effects on the soil microbial biomass. The soil microbial biomass increased with added FOM, reached a maximum, and then declined as the FOM decomposed. The FOM had a more significant stimulating effect on microbial biomass with water addition. Under the soil moisture ranges used in this experiment (21.0%–29.7%), FOM input was more important than water addition in the soil C mineralization process. We concluded that short-term FOM input into the belowground soil and water addition do not affect the SOC pool in shrubland in an arid region.

## Introduction

Soils contain more carbon (C) than the atmosphere and biomass [1]. Approximately 75–100 Pg C yr^−1^ is released from this large C pool [2], and it has been estimated that this has increased by 0.1 Pg C yr^−1^ in the last twenty years [3]. Given the large amounts of C in, and released by, soil, the soil's response to environmental change will play a key role in determining future concentrations of atmospheric CO_2_ [4]. It is worth examining the possibility of transferring CO_2_ from the atmosphere to terrestrial C storage as a possible way of restricting increases in atmospheric CO_2_ concentrations and the resulting global warming. Generally, elevated CO_2_ is assumed to stimulate primary production and increase C input to soil [5], and more soil C sequestration is anticipated. However, as warming can also accelerate soil C decomposition, the effects of climate change on the soil C pool remain uncertain [6]. Therefore, accurately estimating soil C dynamics is critical for evaluating the potential effects of global climate change on the terrestrial biosphere [7].

An imbalance between the input of plant C and its decomposition rate results in changes in stored soil C [8]. A soil will give net C sequestration from increased C input if the organic C decomposition rate does not increase [9]. However, many studies have concluded that soil C does not necessarily increase when the organic C input from vegetation increases, [5], [10], [11], [12], and have indicated that increasing the C input has a stimulating effect on C decomposition. Fresh organic C input can supply energy and nutrients for the soil microbes, and can, therefore, accelerate soil organic C (SOC) mineralization[13], which has been called a “priming effect” [14]. This process is mainly associated with stimulation of the microbes by the easily decomposable organic matter, and causes an increase in CO_2_ efflux [15]. Fontaine et al. also reported that fresh C input may accelerate soil C decomposition and cause the soil C store to decrease [16]. Sayer et al. found that litter inputs can significantly increase soil C release in a tropical forest, but that doubling litter inputs caused a smaller increase in soil C release than adding less than 30% litter [12]. The relationship between organic C input and soil C sequestration is, therefore, quite uncertain.

Besides C input, soil moisture is also an important factor affecting ecosystem C exchange and soil organic matter decomposition [8], [17]. Water availability can regulate plant growth and net ecosystem productivity [18], and, therefore, affects C input to the soil. Water limitations in arid and semiarid ecosystems will be exacerbated by global warming and lower soil water availability [19]. Microbial activity requires a certain soil water content range, and is limited by soil continually drying out [20], [21]. Contact between the microbes and the available substrate and the physiological performance of microbes are limited at low soil water contents [22]. Therefore, droughts could reduce the decrease in soil C by inhibiting microbial decomposition. After long-term observations, it has been found that precipitation has significantly increased in eastern America, northern Europe, and central Asia, but has decreased in many other regions [23]. Because precipitation is highly variable, spatially and temporally, specific environmental factors in the area where a study is conducted must be considered.

Although many studies have been conducted on the effects of climate change on the soil C cycle, arid regions have received relatively little attention [24]. Arid regions occupy approximately 20% of the global terrestrial surface, and they are expanding because of climate change, fire, and land-use change [25]. Arid ecosystems have been predicted to be amongst the most responsive ecosystem types to global climate change [26]. The amount of soil C stock in arid ecosystems is huge [27], and has been estimated to include 241 Pg of organic C and a similar or larger amount of inorganic C [1]. The significant potential C sink capacity in arid ecosystem soils using restorative management could make arid regions an important factor in global climate change [27].

The Xinjiang Uighur Autonomous Region (XUAR) in northwest China covers over one-sixth of China's land area and includes the majority of the country's arid areas [28]. This region is characterized by widely distributed saline/alkaline soils and low precipitation [29], [30]. High alkalinity, high salinity [31], and low soil water content [20], [21] can inhibit the activities of microbes, and can therefore decrease soil organic matter decomposition. Soils may receive more plant C input because of increased primary production under elevated CO_2_ and increased precipitation. Therefore, we wondered whether these soils could sequester more C because of global warming. If soils sequester more C, large areas of XUAR will become a huge C sink, and could partly offset global warming. Precipitation in the arid regions of central Asia is likely to increase significantly in the next 50 years [23], [32]. However, the relationship between C sequestration and increased precipitation in arid regions is not clear. Identifying the response of soil C to increased plant C inputs and precipitation would help us to gain a better understanding of C cycling in arid regions under global climate change. We applied plant litter inputs and water addition to soil to simulate the main effects of climate change on soil C cycling in an arid region of northwest China. Specifically, we aimed to address the following questions: (1) what are the effects on the soil C pool of increased plant litter input, increased precipitation, and their interaction? and (2) how do microbes change seasonally under increased plant litter inputs and water addition?

## Materials and Methods

### Ethics Statement

All necessary permits were obtained for the field studies described. The study sites are managed by the Fukang Station of Desert Ecology, Xinjiang Institute of Ecology and Geography, Chinese Academy of Sciences.

### Study site description

The study was conducted at the Chinese Academy of Sciences' Fukang Station of Desert Ecology (87° 56′ E, 44° 17′ N, elevation 461 m), which is located at the southern edge of the Junggar basin, in the hinterland of northwest China. The region has an arid climate with mean annual rainfall of 160 mm and a mean annual temperature of 6.6°C [29]. Soil is clay-loam textured with heavy salinity/alkalinity.

The field site is shrubland, and canopy coverage is below 40%. It is dominated by *Reaumuria soongorica*, which is the dominant native vegetation species in the XUAR. The main species present include *Haloxylon ammodendron*, *Nitraria sibirica*, *Salsola collina*, *Ceratocarpus arenarius*, and *Suaeda glauca*. The soil physical and chemical properties are shown in Table 1.

**Table 1:** Descriptions of soil physical/chemical properties in the study sites.

Soil depth (cm)	Soil organic carbon (SOC, g/kg)	Soil bulk density (g/cm^3^)	pH	Soil EC (dS/m)
0–10	4.66±0.61	1.29±0.05	9.36±0.12	2.11±0.19
10–20	3.26±0.33	1.29±0.07	9.34±0.17	1.66±0.20
20–30	2.15±0.24	1.39±0.03	9.43±0.34	0.87±0.09

Values represent mean±SE (n  =  5).

### Experimental design

The experiment used a nested design with fresh added organic matter (FOM) and water (P) as the two controlled parameters. In early June 2011, 27 randomly distributed study plots (3 m×3 m) were established in a *R. soongorica* community with uniformly distributed vegetation. Adjacent plots were at least 2 m apart, to mitigate buffering effects.

Spring and autumn are windy in the study region, so it was impossible to add FOM to the soil surface without it being disturbed by the wind. The main root of *R. soongorica* is 0–30 cm deep, and approximately 76% of feeder roots are located above 20 cm [29]. Because the SOC in deep soil layers (> 20 cm) is relatively stable and can only receive extremely small amounts of plant C [16], we chose 20–30 cm as the FOM input layers.

FOM was added to the 20–30 cm deep soil to increase the SOC by 0% (F0, no FOM input), 5% (F1, 33.64 g/m^2^), and 10% (F2, 67.28 g/m^2^). Water addition treatments were initiated at the start of the experiment, and provided different amounts of water each week, based on the long-term mean weekly precipitation records at the nearby weather station at the Fukang research station. At each water addition plot, water was added with the sprinkling can manually. The three water addition treatments were equivalent to 0% (P0, no additional irrigation), 50% (P1, 2.43 mm), and 100% (P2, 4.87 mm) increased precipitation. A randomized-block design was used for the plots, with the three FOM input treatments nested within the three irrigation treatments, and with three replications.

The FOM was plant litter collected from an adjacent *R. soongorica* community site at the end of the growing season 2010. After collection, impurities such as lumps of soil were removed and the plant material was dried at 65°C in an oven for 48 h, and then the material was stored at 4°C until use. Before use in the field experiments, the plant materials were ground and passed through a 2 mm mesh sieve. The C, N content and the C: N of the FOM were 44.42±3.31% (mean±SE, n = 5), 3.18±0.08% (n = 5) and 13.94±0.97 (n = 5), respectively.

To prepare the plots, we first collected the dead plant tissues and litter from the soil surface (a very small amount), and these were replaced after the initial plot treatments had been completed. Next, we carefully removed the top 0–20 cm soil in layers, taking care to keep the soils in their original shape and structure, and ensuring that the plants and roots were not badly affected. The FOM was then added. Finally, the top soil was replaced in its original position, and gaps between the soil blocks were filled with soil from the 0–10 cm layers collected from an adjacent area. The F0 plots were treated the same as the added FOM treatment plots. Adding the FOM to the upper soil layers was difficult without damaging the root systems [11]. The canopy radius of *R*. *soongorica* near the study site was 0.26±0.05 m, and the root biomass out of this range was about 25% of total root biomass. For avoiding drastically damage the root systems, soil under the plant canopy was not removed.

To evaluate the effect of soil disturbance on treatments, we chose extra three natural plots adjacent experimental site, and soil respiration was measured on natural plots and treatment plots with an LI-8100 automated soil CO_2_ flux system from 8:00 to 20:00 in two-hour rounds at the first sampling time in late June and July (with high soil moisture). The results showed that no significant difference was found between the soil respiration data on the treatment plots and natural plots (*P*>0.05). The mean soil moisture for the natural and F0P0 plots were 21.54±0.10% (v/v) and 21.06±0.11% (v/v) in June, and 24.01±0.25% (v/v) and 23.86±0.21% (v/v) in July, respectively. As the soil respiration significantly correlated with microbial and root biomass [8], the impacts by the treatments on soil could be limited.

### Root biomass measurements

Root biomass was determined in soil cores. Because the shrub roots were highly spatially variable, root samples were collected from one large square core (25 cm×25 cm, and 60 cm deep) from each plot (27 plots) in October 2011, and separated into 0–20 cm, 20–40 cm, and 40–60 cm layers. The roots were carefully separated from these samples, retaining only apparently living material based on the color, texture, and shape of the roots. Fine roots were classified by their diameter (<2 mm). All roots were dried at 70°C to a constant mass and weighed. We excluded roots deeper than 60 cm because very few roots reach that depth.

### Soil temperature and moisture measurements

The soil temperature (°C) and soil water content (v/v, %) at 5, 15 and 25 cm depths of each single irrigation treatments (F0P0, F0P1 and F0P2) were monitored using sensor (ECH_2_O EC-TM, Decagon Devices, Inc. Pullman, WA USA). Outputs of each probe was adjusted with soil collected near the plot where the probes would be equipped, using the method described by Starr and Paltineanu [33], and there were no significant difference for outputs of used probes in same soil in this experiment. Means of data at three depths were taken as soil temperature and moisture of 0–30 cm depth.

### Analysis of soil physical and chemical properties

The first soil sampling was carried out in late June (20 days after experiment setup) for reestablishment of the soil microbial community. Soil samples were collected from 0–30 cm deep. Three samples were taken from each of the three replicate plots and mixed to make three samples for analysis for each plot treatment. Sampling was performed monthly from June to October. Each sample was passed through a 2 mm sieve immediately and separated into two parts, one of which was stored at 4°C for microbial biomass C (MBC) analysis and the other naturally dried at room temperature. MBC was determined using the fumigation-extraction method. The pH (using a 1 5 soil: water ratio) and electrical conductivity (EC; using a 1 5 soil: water ratio) were determined with a Eutech PC700 pH/EC meter (Thermo Fisher Scientific Inc., Waltham, Massachusetts, USA). SOC was measured using the method described by Nelson and Sommers [34].

### Data analysis

Linear regression analysis, ANOVA, and homogeneity of variance tests were performed using SPSS 13.0 software [35]. Multiple comparisons of means for different treatments were analyzed using Tukey's test.

## Results

### Temperature and soil moisture

**Figure 1:**
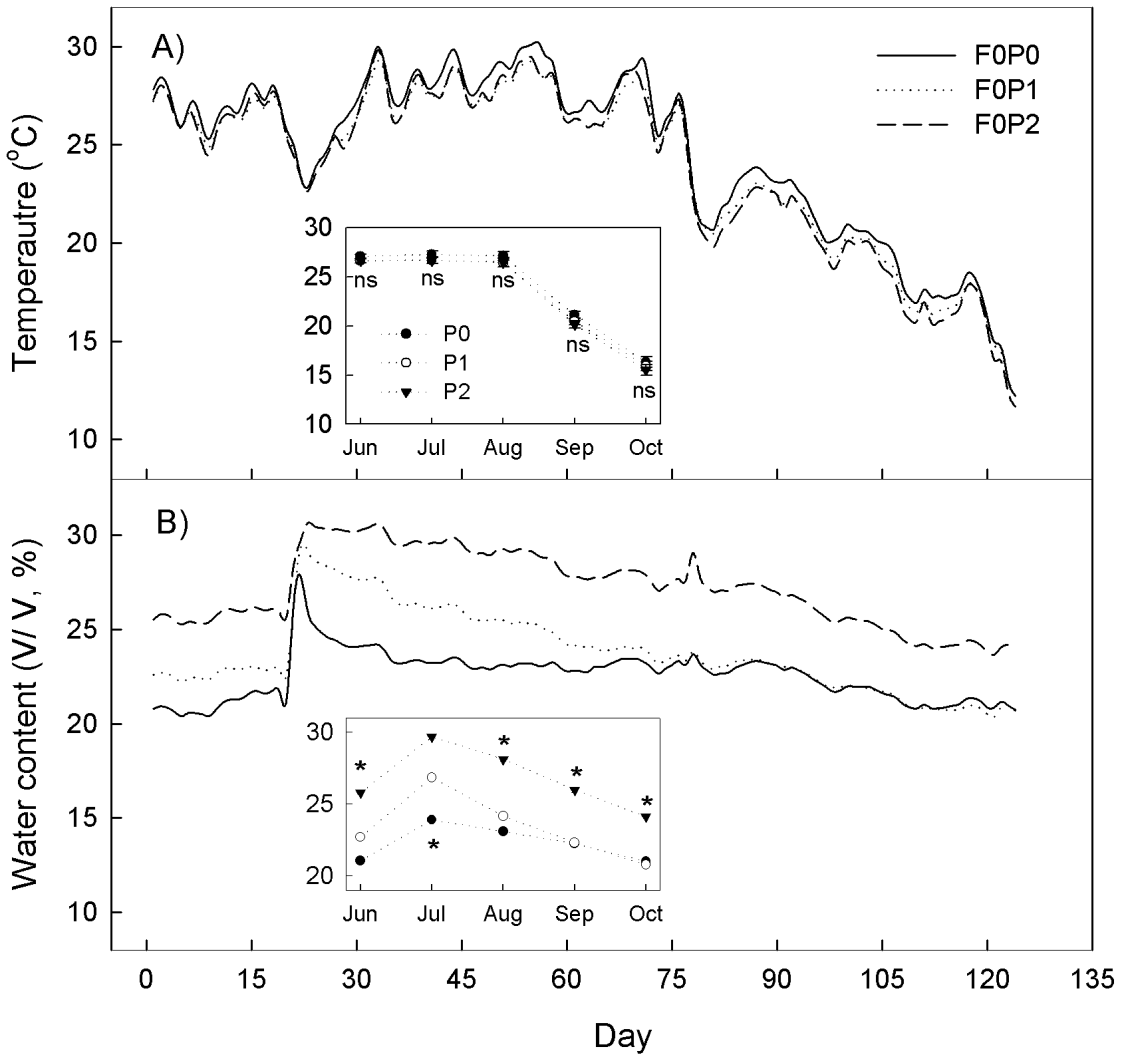
Dynamics of Temperature (A) and soil moisture (B) under different water addition regimes without FOM treatments during the study period. Inserts represent the monthly mean values. The day 0 represents the first sampling time. FOM: fresh organic matter; F0: no FOM input; P0, P1, P2: no added water, 50% and 100% increase in precipitation, respectively. * means significant (*P*<0.001), and ns mean not significant (*P*>0.05) among the three water addition levels in the same month.

During the study period (June–October), water addition treatments had no significant effects (*P*>0.05) on soil temperature, although we did observe slightly lower temperatures in the plots with added water. However, water addition significantly increased soil moisture (*P*<0.001, Figure 1), by an average of 1.1% and 4.5% (v/v) for the P1 and P2 water addition regimes, respectively. When analyzed monthly, soil moistures in the P1 and P2 plots were significantly higher than the P0 plots for the first 3 months (June–August), then the soil moisture in the P0 and P1 plots were similar for the last two months (September and October). The amount of rain at day 21 was 28 mm, and increased the soil moisture over 5% (Figure 1). Under the P0, P1, and P2 water addition treatments, the monthly mean soil temperature ranged from 16.31±0.55°C (mean±SE) to 27.08±0.19°C, 15.93±0.48°C to 26.61±0.18°C, and 15.51±0.54°C to 26.65±0.21°C, respectively, and the soil moisture values ranged from 21.00±0.05% to 21.06±0.11%, from 20.77±0.05% to 22.70±0.06%, and 24.10±0.06% to 25.77±0.07% (v/v), respectively.

### SOC

**Figure 2:**
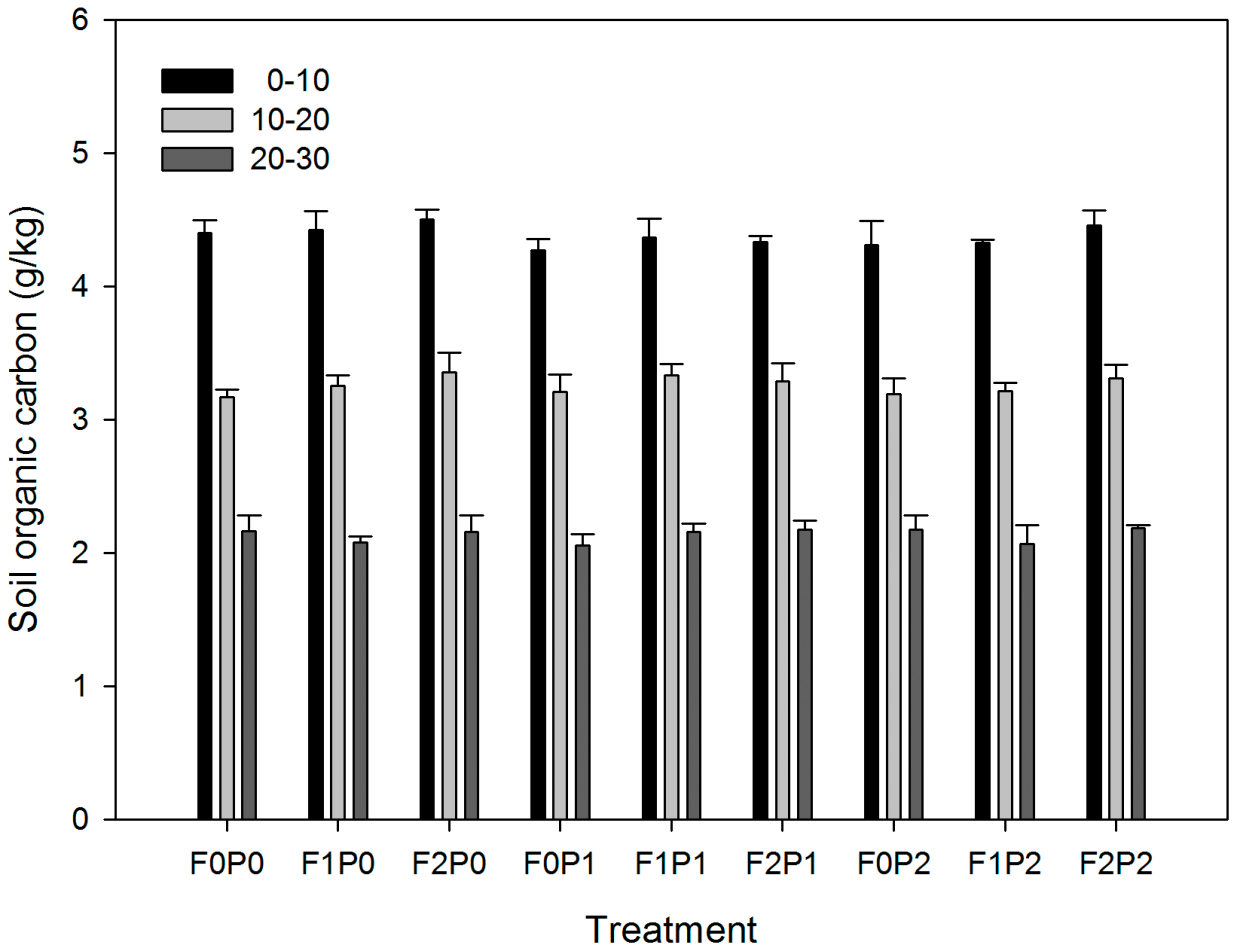
Final levels of soil organic carbon (mean±SE) in response to added precipitation and fine particulate matter after five months of manipulation. F1 and F2: 5% and 10% increase in SOC. Other abbreviations are same as Figure 1.

The input of FOM, water addition, and their interactions had no significant effects on the SOC. SOC content declined significantly with depth, and approximately 76% of the SOC in the soil from 0–30 cm deep was found above 20 cm. However, the interactions between FOM, water addition, and soil depth had no significant effects on the SOC. The SOCs in the F2 treatment soils were slightly higher than in soils from the same layers in the other treatment plots, but the differences were not significant (Figure 2).

### Microbial biomass C

**Figure 3:**
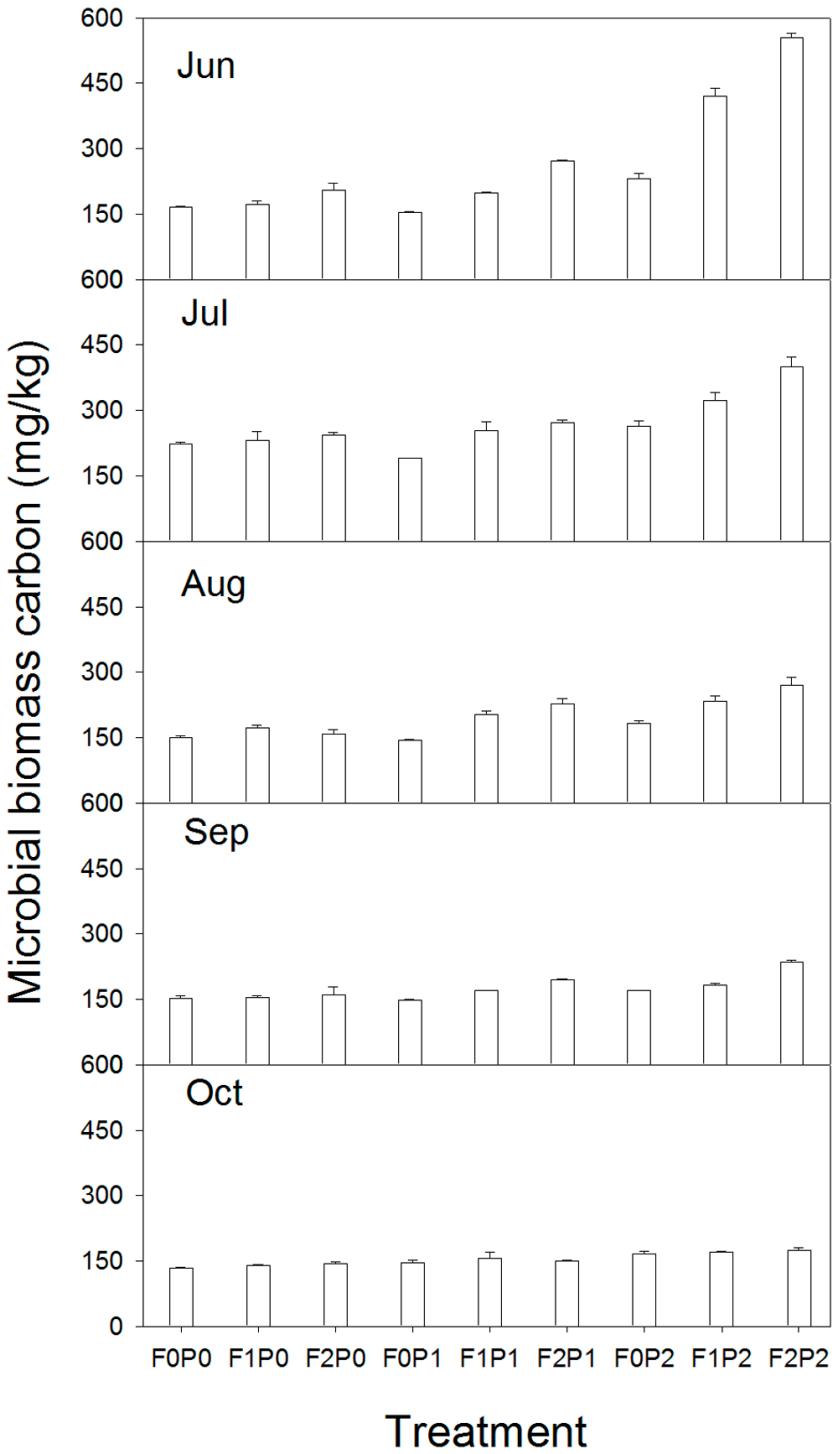
Seasonal dynamics (mean±SE) of microbial biomass carbon under different treatments. Other abbreviations are same as Figure 1.

The FOM inputs, water addition, and their interactions had significant effects on the soil MBC (*P*<0.001, Table 2). The soil MBC content was at its highest at the start of the experiment, in June, and then declined with time. The soil MBC content was 4.6%–68% lower in the same treatment plots at the end of the experiment (October) than that at the beginning (Figure 3).

**Table 2:** Results (*F*-Values) of ANOVA with fresh organic matter (FOM), water addition and their interactions on soil organic carbon (SOC) (g/kg), microbial biomass carbon (MBC) (mg/kg) and fine root biomass (g/m^2^, above 60 cm soil depth).

	SOC		MBC		Fine root biomass	
Source	df	*F*	df	*F*	df	*F*
FOM (F)	2	1.80^ns^	2	176.20^**^	2	9.68^ **^
Water addition (P)	2	0.27^ns^	2	342.73^**^	2	21.29^**^
F×P	4	0.46^ns^	4	35.37^**^	4	1.20^ ns^

ns, * and ** represents *P*>0.05, *P*<0.05 and *P*<0.001, respectively.

**Figure 4:**
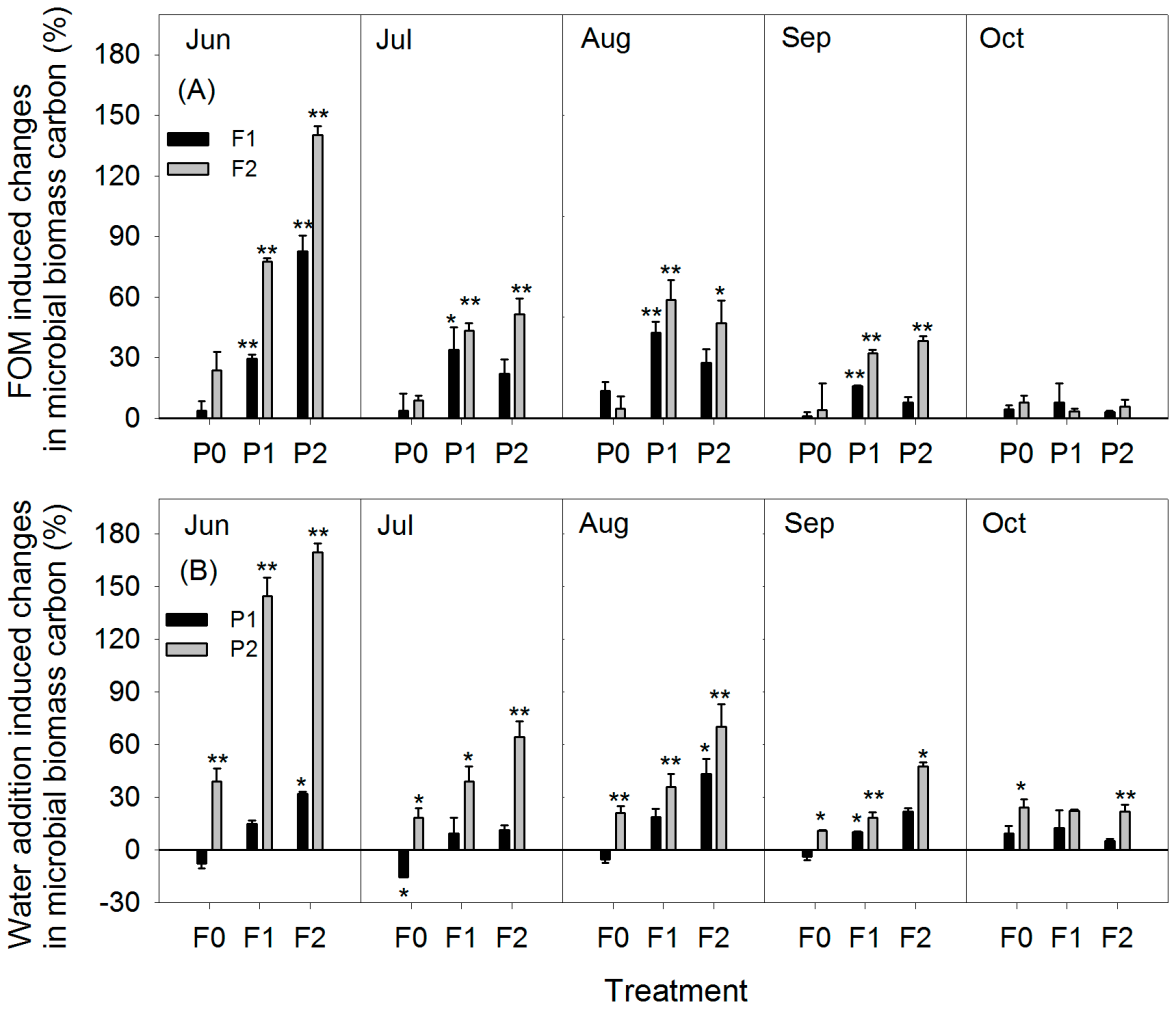
FOM (A) and water addition (B) induced changes in microbial biomass carbon (mean±SE). * and ** represent significant difference compared with controls at *P*<0.05 and *P*<0.01. Other abbreviations are same as Figure 1.

The FOM-induced increase in MBC ranged from 3.7% to 140.2%, 3.6% to 51.4%, 4.7% to 58.8%, 1.1% to 38.4%, and 3.0% to 7.8% in June, July, August, September, and October, respectively. FOM had no significant effect on the MBC in the treatments without water addition. The increase in MBC caused by water addition ranged from −7.9% to 169.7%, −15.4% to 64.5%, −5.3% to 70.1%, −3.8% to 47.5%, and 5.0% to 24.2% in June, July, August, September, and October, respectively (Figure 4).

The Pearson correlations were not significant between the MBC and monthly mean soil moisture for all treatments (P>0.05).

### Fine root biomass

FOM input and water addition had significant effects on the fine root biomass separately (*P*<0.001), but their interactions did not (*P*>0.05, Table 2). Fine root biomass was greatest in the upper 0–20 cm of the soil and decreased significantly with soil depth.

**Figure 5:**
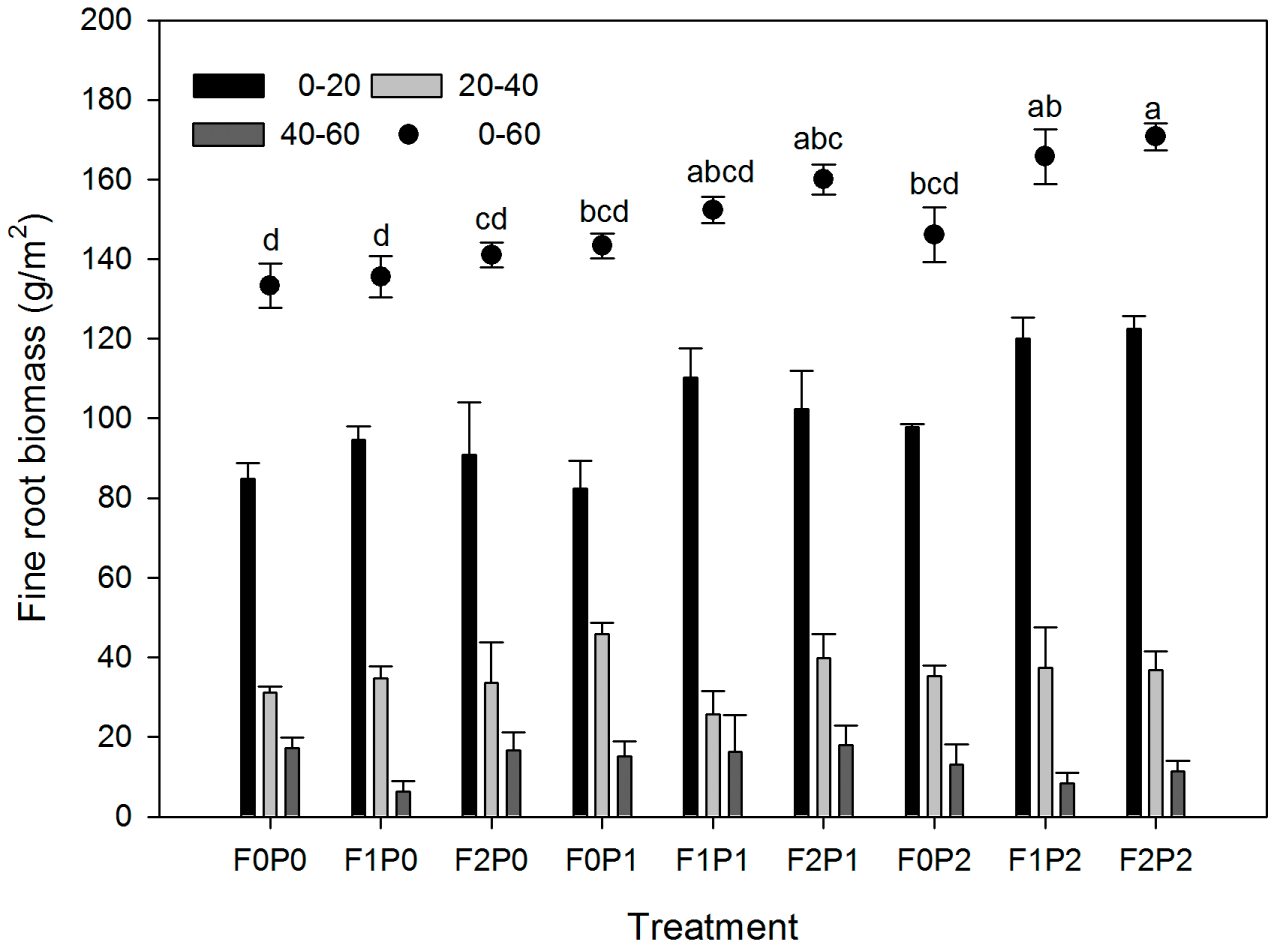
Effects of FOM and water addition on fine root biomass (mean±SE). Dots with different lowercase letters are significantly different from each other of fine root biomass in 0–60 cm soil layers in the different treatments at *p*<0.05 (Tukey Test). Other abbreviations are same as Figure 1.

**Figure 6:**
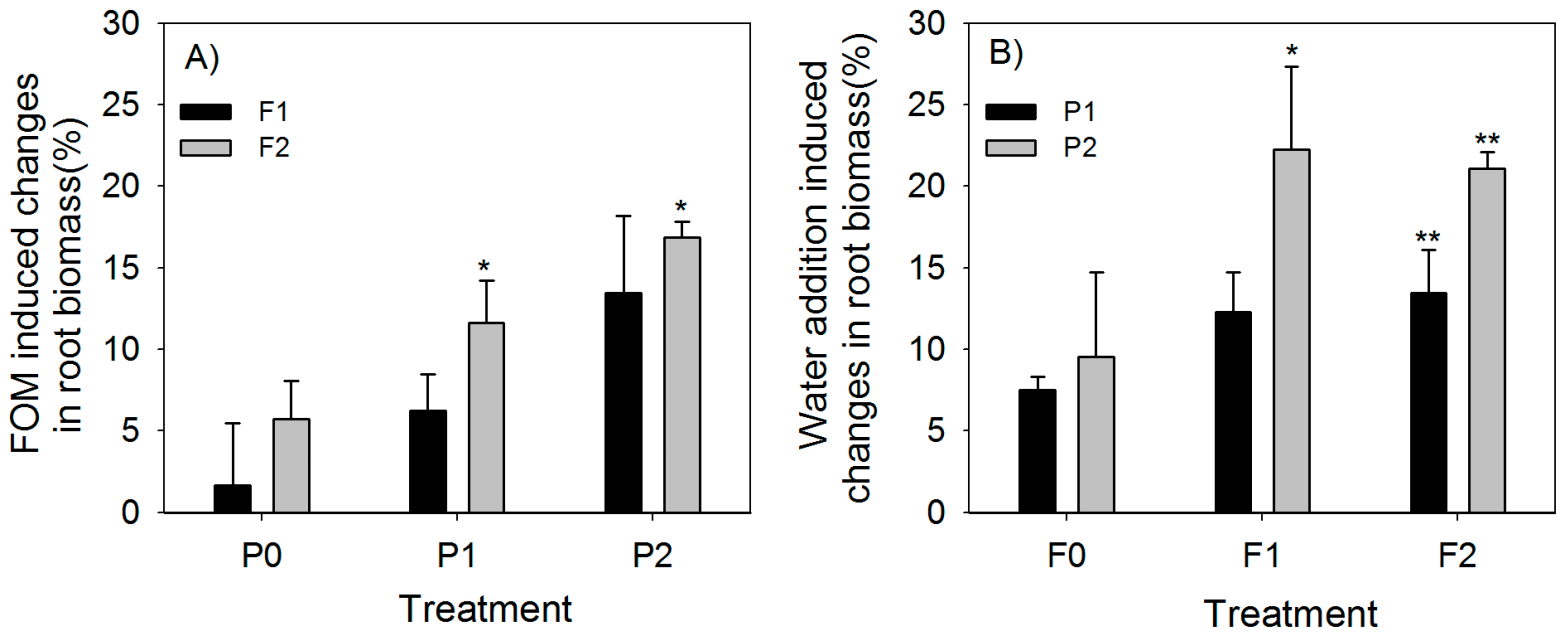
FOM (A) and water addition (B) induced changes in fine root biomass (mean±SE). * and ** represent significant different compared with controls at *P*<0.05 and *P*<0.01. Other abbreviations are same as Figure 1.

Fine root biomass between 0 and 60 cm deep ranged from 133.3±5.6 g/m^2^ in the F0P0 treatment to 170.8±3.4 g/m^2^ in the F2P2 treatment. There were few significant differences in fine root biomass between the treatments with no FOM inputs or no water addition (Figure 5). Under the P1 and P2 water addition regimes, increased FOM inputs caused significant increase of fine root biomass (Figure 6A). Similar results were found for water addition (Figure 6B). Water addition increased the root biomass much more than FOM input did (Figure 6).

## Discussion

### Effects of FOM input on soil C

Soil organic matter plays a key role in determining soil quality and preserving plant nutrients [7]. In the natural environment organic matter inputs to the soil are supplied by plants. Although the response of the soil organic pool to climate change is quite uncertain [16], many laboratory-based incubation and field experiments have provided evidence for changes in SOC storage with increased soil organic matter. After supplying fresh C into soil, Fontaine et al. observed a faster soil C decomposition rate and a decrease in soil C content [36]. Xiao et al. reported that SOC changed slightly with C inputs in a temperate grassland [11]. Other authors have stated that soil C does not necessarily increase with increased organic C input [10], [12]. Similarly, in this experiment, no remarkable changes in the SOC in the same soil layers were found from the different FOM input regimes. During the experimental period nearly all FOM input to the soil decomposed, regardless of the water content, and did not affect the SOC in other layers that didn't have direct FOM input. Trumbore and Czimczik stated that because it is physically isolated from microbes, soil organic matter can persist at a steady level in soil, whereas FOM input to soil is more easily decomposed and can be the major source of C loss from soils [7]. This is likely to be a reason for the steady SOC levels at the end of our experiment. We also have to consider that in the natural environment plant litter only drops onto the soil surface, and cannot be fully accessed by the microbial decomposers. This aboveground C input seems not to be an effective way of promoting soil C sequestration, at least in the short-term [9].

The SOC stock in soils results from the balance between the C inputs and outputs [6]. Plants supply the organic matter inputs, which are then transformed by the soil fauna and microorganisms [13]. As decomposers of SOC, microorganisms have received much interest because of their important role in the dynamics of the soil C pool [7], [20], [27]. MBC increased remarkably with increased FOM compared with the other treatments at the start of our experiment (Figure 4A). This may indicate that the soil system is resource limited, which is the case in most soil systems [37]. After the initial increase, a large decrease in MBC was found in most treatments, and no significant differences were found between treatments with the same water addition regimes in the last month of the study (Figure 4A). Seasonal MBC decreases have been observed by other authors, and this can partly be attributed to changes in environmental factors or plant physiology [37], [38].

Fine roots are usually defined as those with diameters<2 mm. Although the amounts of these make up a small proportion of the total root biomass, they can make a major contribution to C and nutrient input to the soil [39]. Nutrient-rich FOM input can affect plant growth because it can lead to an increase in nutrient availability in the soil [40]. There have been a few reports showing different fine root responses to organic C input or measures that affect organic C input (e.g., ground litter addition or removal) in several ecosystems [11], [41], [42], and significant effects of FOM input on the fine root mass was found in this experiment.

### Effects of water addition on soil C

Soil water availability can regulate root growth and the performance of microbes, and can indirectly affect the belowground soil C cycle [18], [21]. Consistent with previous studies [19], [43], this study showed that water addition plays an important role in belowground C stock in arid land with low precipitation. Although water addition had little effect on the SOC, the treatments did significantly affect the MBC and fine root growth.

Seasonal variability of the soil microbial biomass is important for soil nutrient release and mineralization [37], and it closely correlates with precipitation [44]. Some studies have reported that microbial biomass is highest in dry periods and lowest in rainy periods, and have attributed the reduced microbial biomass and increased turnover of microbes to enhanced microbivory and nutrient competition from plants in wet periods [44], [45]. In contrast to those results, we found significant positive responses of MBC to water addition both with and without FOM input treatments in the first four months (Figure 4B). This can be explained because, in arid regions, soil solute availability is reduced by the low water content, and this is likely to reduce substrate availability to the microbes [37]. The soils in the study area are highly saline and alkaline, and this also has strong negative effects on microbial biomass [31]. The osmotic stress caused by the salinity and alkalinity of the soils would have been relieved by water addition. The microbial biomass, therefore, increased in the irrigated plots.

Continuous absorption of water is essential for the growth and survival of most plants because of their daily water consumption. In arid ecosystems, soil moisture is generally considered to be the primary factor limiting root growth, especially for desert shrubs, which are often exposed to long periods of drought [46]. In this study, water irrigation showed a significant stimulating effect on the fine root biomass. This was similar to studies in other water limited ecosystems [46], [47]. In the treatments without FOM inputs, more water addition caused an approximately 10% increase in fine root biomass (Figure 6B). These results indicate that significantly increased precipitation in the arid regions of central Asia would stimulate plant root growth and indirectly affect soil C sequestration [32].

### Interactive effects of FOM input and water addition

Given that both FOM input and water addition showed positive effects on the belowground C cycle, their interactive effects were assumed to be larger still. FOM input caused larger changes in MBC in the treatments with water addition than in those without water addition (Figure 4A). Similar trends were also found for changes in the MBC caused by water addition (Figure 4B). Soil in the arid climate region only receives a small amount of plant litter input because vegetation is sparse and water is limited [27], [48]. Increases in soil microbes are limited by the substrate and the lower soil solute availability [37]. The interactive effects of the two treatments, therefore, caused larger changes in MBC than the single treatment did.

The root biomass is closely correlated with soil moisture and nutrient availability [41], [42]. Wilcox et al. stated that soil moisture is the primary factor influencing root growth in arid ecosystems, and other factors, such as nutrients and soil temperature, are secondary limiting factors [46]. After comparing the single factor effects on fine root biomass, water addition played the most important role in the root growth process in our study (Figure 6). After the soil moisture was increased by adding water, FOM inputs also caused significant increases in fine root biomass. As mentioned above, FOM decomposition through microbial activity was inhibited at low soil moisture contents, which led to weaker effects from FOM input on fine root growth, in contrast to the water addition effects.

### Possible mechanism: function of microbes

The microbial biomass plays a key role in the processes that lead to priming effects [15]. Because of comparatively rapid changes in microbial biomass and detectable differences in the amount of biomass present, it can be used as a sensitive indicator of soil SOC variations [44]. Therefore, measuring microbial biomass is an effective way of understanding soil C pool dynamics associated with climate change.

Fountain et al. found that adding ^13^C labeled organic matter significantly increased microbial biomass, and that the increase mainly came from the formation of labeled biomass [36]. After the supplied organic C was exhausted, many specialized microbes died or became dormant because they were unable to use SOC [36]. In our study, the differences in microbial biomass caused by adding FOM were smaller in August to October than those in June and July. These changes might be due to the exhaustion of FOM, leading to a decrease in the population of microbes that used this resource.

At the end of the experiment, we found that FOM input did not enhance soil C accumulation. A similar result was reported by Xiao et al. in a semi-arid grassland [11]. This might be caused by the FOM inducing only a weak priming effect that did not influence SOC mineralization, and by most of increase in microbial populations induced by the FOM input being only FOM decomposing microbes that cannot survive on SOC [13], [16]. Further, soil organic matter (SOM) is predominantly stabilized in the microaggregates, which can protect SOM to avoid being easily mineralized by microbes [49]. This could be another reason for the stabilization of the SOC.

## Conclusions

We found minor changes in the SOC pool caused by adding FOM and water. Adding FOM had a stronger stimulating effect on the soil C mineralization process than did adding only water. However, adding water had an important indirect effect on the decomposition of organic matter, fine root growth, and microbial biomass dynamics. Research is required on the nature of the plant litter decomposition process in the field. These results increase our understanding of how soil C dynamics may change with global climate change.
